# Satellite cell response to concurrent resistance exercise and high-intensity interval training in sedentary, overweight/obese, middle-aged individuals

**DOI:** 10.1007/s00421-017-3721-y

**Published:** 2017-10-25

**Authors:** Jamie K. Pugh, Steve H. Faulkner, Mark C. Turner, Myra A. Nimmo

**Affiliations:** 1 School of Sport, Exercise and Health Sciences and National Centre for Sport and Exercise Medicine, Loughborough University, Loughborough, Leicestershire LE11 3TU UK; 2 College of Life and Environmental Sciences, University of Birmingham, Edgbaston, Birmingham B15 2TT UK; 3 Department of Engineering, School of Science and Technology, Nottingham Trent University, Nottingham, NG11 8NS UK

**Keywords:** Concurrent exercise, Resistance exercise, High-intensity interval training, Obesity, Acute responses, Interference, Satellite cell, mRNA expression, Human skeletal muscle

## Abstract

**Purpose:**

Sarcopenia can begin from the 4–5th decade of life and is exacerbated by obesity and inactivity. A combination of resistance exercise (RE) and endurance exercise is recommended to combat rising obesity and inactivity levels. However, work continues to elucidate whether interference in adaptive outcomes occur when RE and endurance exercise are performed concurrently. This study examined whether a single bout of concurrent RE and high-intensity interval training (HIIT) alters the satellite cell response following exercise compared to RE alone.

**Methods:**

Eight sedentary, overweight/obese, middle-aged individuals performed RE only (8 × 8 leg extensions at 70% 1RM), or RE + HIIT (10 × 1 min at 90% HR_max_ on a cycle ergometer). Muscle biopsies were collected from the vastus lateralis before and 96 h after the RE component to determine muscle fiber type-specific total (Pax7^+^ cells) and active (MyoD^+^ cells) satellite cell number using immunofluorescence microscopy.

**Results:**

Type-I-specific Pax7^+^ (*P* = 0.001) cell number increased after both exercise trials. Type-I-specific MyoD^+^ (*P* = 0.001) cell number increased after RE only. However, an elevated baseline value in RE + HIIT compared to RE (*P* = 0.046) was observed, with no differences between exercise trials at 96 h (*P* = 0.21). Type-II-specific Pax7^+^ and MyoD^+^ cell number remained unchanged after both exercise trials (all *P* ≥ 0.13).

**Conclusion:**

Combining a HIIT session after a single bout of RE does not interfere with the increase in type-I-specific total, and possibly active, satellite cell number, compared to RE only. Concurrent RE + HIIT may offer a time-efficient way to maximise the physiological benefits from a single bout of exercise in sedentary, overweight/obese, middle-aged individuals.

## Introduction

Ageing often results in the degenerative loss of significant muscle mass and strength, known as sarcopenia (Bijlsma et al. 2013), a process starting as early as the 4th or 5th decade (Marcell 2003; Jackson et al. 2012). Exercise can lessen the effect of sarcopenia, however, 45% of women and 33% of men do not meet the current physical activity guidelines (Health and Social Care Information Centre 2013). Sarcopenia is further accelerated in the presence of obesity and can result in physical disability and a lower quality of life (Dominguez and Barbagallo 2007; Stenholm et al. 2008) and in England, 58% of women and 65% of men are classified as overweight or obese (Health and Social Care Information Centre 2016). The combination of physical inactivity and obesity underpins a number of chronic diseases (e.g., type 2 diabetes and cardiovascular disease) (Rana et al. 2007; Reddigan et al. 2011), and are considered major global public health issues. Strategies to encourage increased physical activity in these populations, which may in turn reduce obesity, could slow the aging process and development of chronic disease.

Exercise is an effective stimulus for inducing increases in muscle mass, weight loss and cardio-metabolic health irrespective of age, and therefore, could play a major role in combatting the fight against the increase in obesity and obesity-related diseases. Current exercise guidelines recommend that middle-aged individuals (~ 40 to 65 years) should engage in a combination of endurance and resistance exercise (RE), improve cardio-metabolic health and quality of life (Chief Medical Office 2011; Garber et al. 2011). It is often recommended that individuals should complete five 30 min sessions of moderate-intensity endurance exercise and two sessions of RE per week, therefore, requiring up to 7 days of exercise engagement per week, which may provide a significant barrier to some.

The design of a concurrent training program incorporating RE and endurance exercise within a single session provides a practical, time-efficient protocol that may be more appealing to individuals, particularly those who are not “natural exercisers”, and therefore, increase motivation and adherence. In support of this viewpoint, Larose et al. (2012) implemented a 6 month concurrent training program with sedentary, overweight/obese, middle-aged individuals with type 2 diabetes. The participants were given the option as to when they performed each exercise component. Remarkably, all participants chose to perform both RE and endurance exercise components within a single session. However, there is evidence to suggest that combining RE and endurance exercise will impair strength development and muscle size (Hickson 1980; Craig et al. 1991; Hennessy and Watson 1994; Coffey et al. 2009a, b; Babcock et al. 2012; Kikuchi et al. 2016; Fyfe et al. 2016), although such evidence is equivocal (Bell et al. 1991; Shaw et al. 2009; Donges et al. 2012; Lundberg et al. 2012, 2013; Apró et al. 2013; Kazior et al. 2016). We have recently shown that using an acute bout of high-intensity interval training (HIIT) in combination with RE, as an alternative to moderate-intensity endurance exercise with RE, does not impede acute (< 6 h) gene expression and protein signalling markers of muscle growth compared to a single bout of RE alone in young, healthy individuals (Pugh et al. 2015). Furthermore, concurrent RE + HIIT resulted in greater increases in the expression of PGC-1α mRNA suggesting parallel endurance-type adaptations (Olesen et al. 2010). Thus, an exercise protocol that combines both RE and HIIT, as an alternative form of endurance exercise, into a single session, may help individuals meet current exercise guidelines in a time-efficient manner without compromising RE and endurance exercise-induced adaptations. Although this evidence provides indicative responses, it is unlikely that the mechanism behind the impaired adaptations following concurrent training can be fully explained by the initial molecular interference between the signalling proteins, AMPK and mTOR (Hamilton and Philp 2013).

Satellite cell content has been shown to be correlated with an improvement in cross-sectional area of the quadriceps muscle following RE training (Bellamy et al. 2014). Utilising this methodology, following an acute concurrent exercise protocol of RE plus moderate-intensity endurance exercise (Babcock et al. 2012) demonstrated an impairment in satellite cell response after concurrent exercise (−6% change from baseline) compared to RE only (38% increase) in young, healthy males. This disparity in the satellite cell response was reported to occur in a fiber-type-specific manner. There was a suppression in type I muscle fiber-specific satellite cell density 4 days (96 h) after both endurance exercise (−7%) and concurrent exercise (−8%), compared to RE only (46% increase). In type II muscle fibers, satellite cell density remained unchanged after endurance exercise and concurrent exercise while increasing after RE only. The authors concluded that concurrent exercise, comprising RE and moderate-intensity exercise, impairs the acute satellite cell response to single bout of RE, thus implicating that this combination could impede muscle growth.

While an increase in satellite cell response to RE is widely accepted (Crameri et al. 2004, 2007; Dreyer et al. 2006; O’Reilly et al. 2008; McKay et al. 2009; Mikkelsen et al. 2009) the response to endurance exercise is limited and inconclusive. Evidence suggests that exercise intensity, rather than duration, may play a key role in the expansion of the satellite cell content (Kurosaka et al. 2012) with studies using moderate-intensity endurance exercise finding no changes in the satellite cell content (Snijders et al. 2011), whereas, studies using high-intensity exercise have shown an increase (Charifi et al. 2003; Verney et al. 2008; Nederveen et al. 2015). Based on these findings, together with our previous study (Pugh et al. 2015), the present study investigated the fiber type-specific satellite cell response to a single bout of RE immediately followed by a bout of high-intensity interval cycling compared to RE alone in sedentary, overweight/obese, middle-aged individuals. It was hypothesised that there would be no interference in the muscle fiber type-specific satellite cell response when a single bout of HIIT is performed immediately after RE.

## Methods

### Participants

**Fig. 1:**
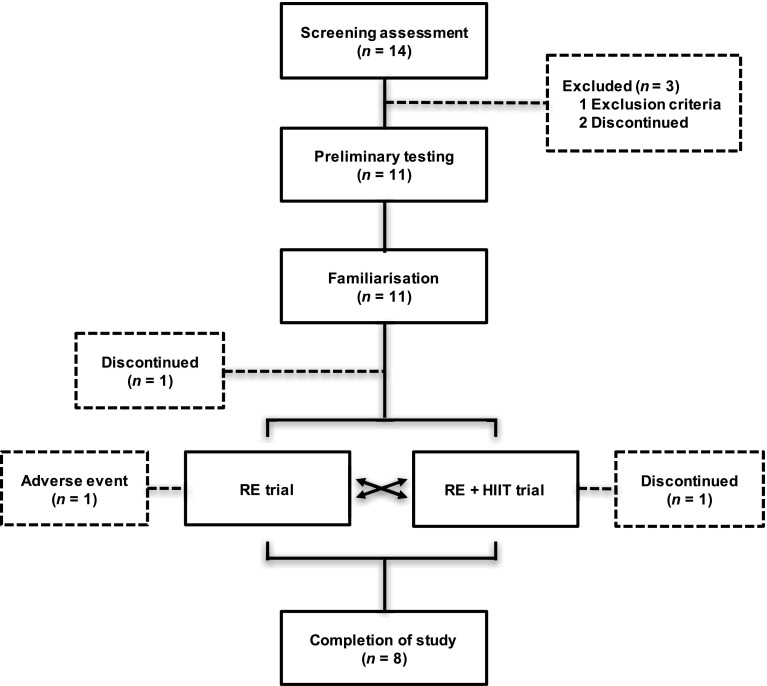
Participant flow diagram. The dashed box indicates the participants who withdrew from the study. Following screening one was excluded because they did not meet the criteria. Three participants withdrew prior to the main trials for unknown reasons. One participant discontinued between the exercise visit and 96 h follow-up visit of the participant’s first experimental trial for unknown reasons. One participant withdrew following completion of the first trial and did not progress to the second trial due to restricted leg movement. The participant fully recovered. *RE* resistance exercise trial, *RE + HIIT* resistance exercise and high-intensity interval training trial

Of the 14 participants enrolled in the study, eight sedentary, overweight/obese, middle-aged male (*n* = 3) and female (*n* = 5) individuals completed both trials and were included in the analysis (Table 1). A participant flow diagram is reported in Fig. 1. Sedentary status was defined as no planned or regular patterns of physical activity or exercise on one or more days per week in the preceding 6 months. Overweight/obese classification was based on a BMI between 27 and 35 kg·m^2^, and the presence of abdominal obesity (male ≥ 94 cm; female ≥ 80 cm). All participants provided full written informed consent. Prior to participation, all participants underwent comprehensive medical assessment, including an electrocardiogram and physical examination to confirm that there were no underlying contraindications to exercise and to confirm that all were free from any medication. A capillary blood sample was taken to analyse fasting glucose, triglycerides, total cholesterol and high-density lipoprotein (HDL)-cholesterol (CardioChek, Polymer Technology Systems, Indianapolis, IN, USA). Participants had no history of diabetes, or presence of the metabolic syndrome. Diagnostic criteria for metabolic syndrome were the presence of any three (or more) of the following factors (Alberti et al. 2005): increased waist circumference (male ≥ 94 cm; female ≥ 80 cm); raised triglycerides (≥ 1.7 mmol·L^−1^); reduced HDL-cholesterol (male < 1.03 mmol·L^−1^; female < 1.29 mmol·L^−1^); raised blood pressure (systolic ≥ 130 mmHg; diastolic ≥ 85 mmHg) and/or raised fasting plasma glucose (≥ 5.6 mmol·L^−1^). The local Human Research Ethics Committee approved all study procedures.

**Table 1:** Participants’ characteristics

Measure	All (*n* = 8)	Males (*n* = 3)	Females (*n* = 5)
Age (years)	48.4 ± 3.9	52.0 ± 0.1	47.7 ± 5.7
Height (m)	1.73 ± 0.03	1.82 ± 0.06	1.68 ± 0.02
Mass (kg)	93.0 ± 4.7	103.4 ± 8.4	86.8 ± 5.6
BMI (kg·m^2^)	30.8 ± 0.9	31.2 ± 0.7	30.6 ± 1.4
Waist circumference (cm)	97.3 ± 2.9	105.3 ± 6.1	92.5 ± 2.6
Systolic BP (mmHg)	120 ± 6	126 ± 9	116 ± 7
Diastolic BP (mmHg)	75 ± 3	75 ± 1	76 ± 4
Glucose (mmol·L^−1^)	5.6 ± 0.3	6.3 ± 0.4	5.1 ± 0.1
Total cholesterol (mmol·L^−1^)	5.34 ± 0.41	4.88 ± 0.30	5.61 ± 0.59
HDL-cholesterol (mmol·L^−1^)	1.57 ± 0.10	1.36 ± 0.24	1.69 ± 0.07
Triglycerides (mmol·L^−1^)	1.22 ± 0.13	1.15 ± 0.17	1.26 ± 0.19
$\dot {V}$O_2 peak_ (mL·kg¯^1^·min^−1^)	25.7 ± 2.6	33.6 ± 4.5	20.9 ± 1.5
Leg extension 1RM (kg)	40.6 ± 5.2	57.1 ± 2.1	30.8 ± 3.5

Data presented as mean ± SEM

*1RM* one-repetition maximum, *BMI* body mass index, *BP* blood pressure, *HDL* high-density lipoprotein, $\dot {V}$ *O*_*2 peak*_ peak oxygen uptake

### Study design and rationale

**Fig. 2:**
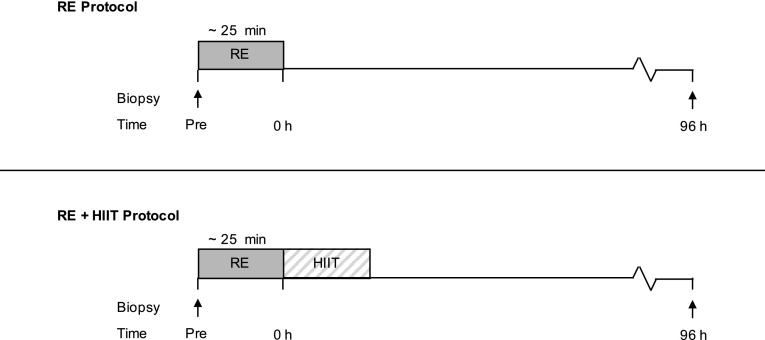
Schematic diagram of the experimental trials. This study adopted a counterbalanced crossover design where participants completed both exercise trials on separate occasions. *RE* resistance exercise trial, *RE + HIIT* resistance exercise and high-intensity interval training trial. Arrows indicate sampling time points for muscle biopsies

A schematic of the study design is displayed in Fig. 2. This study adopted a counter-balanced crossover design. In one session participants completed a single bout of resistance exercise (RE) and in the other session participants performed RE followed by a single HIIT session (RE + HIIT), each trial was separated by a minimum of 14 days (range 14–36 days), during which time the participants were instructed to maintain their habitual lifestyle. Preliminary tests (maximal strength and $\dot {V}$O_2peak_ test) were completed followed by a separate session where participants were familiarised with the RE and HIIT sessions at least 2 weeks before the first experimental trial.

The current project was designed to determine if HIIT performed immediately after RE impairs the satellite cell response to RE. The exercise order was chosen to maximise the anabolic response following RE, which has previously been shown to be diminished when endurance exercise precedes RE (Coffey et al. 2009a, b). Whereas, as we have previously shown no initial molecular interference on gene expression and protein signalling markers of muscle growth with concurrent RE followed by HIIT compared to RE alone (Pugh et al. 2015). Skeletal muscle biopsies were taken before and 96 h after exercise to capture the peak in the RE-induced satellite cell content (Martin and Lewis 2012; Snijders et al. 2015). The timing of the biopsies also allowed direct comparison to Babcock et al. (2012), which is the only other known study to investigate the effects of a single bout of concurrent exercise on the satellite cell response. The present study used a realistic exercise program to elicit an exercise-induced satellite cell response, in comparison to other studies (Crameri et al. 2004; Mikkelsen et al. 2009) using extreme workloads that are unfeasible and result in an exaggerated satellite cell response due to muscle damage. While no measure of muscle damage was made in current study, others using a similar workload have shown that the acute satellite response to a single bout of RE correlates with the degree of muscle hypertrophy following training (Bellamy et al. 2014). Therefore, the acute satellite cell response to a single bout of exercise, irrespectively of the stimuli (exercise-/damage-induced), is still relevant to the potential impact of muscle adaptations to concurrent exercise.

### Preliminary testing

#### Maximal strength

Participants were asked to arrive fasted (at least 4 h) and having avoided any strenuous physical activity 48 h before the preliminary tests. Each participant performed a unilateral one-repetition maximum (1RM) on each leg using a leg extension machine (Technogym, Cesena, Italy). Participants were familiarised with the movement and warmed up prior to testing by performing six repetitions (at ~ 40% of estimated 1RM) and four repetitions (at ~ 60% of estimated 1RM) through a full range of motion with 1 min recovery. After each successful lift, 3 min recovery was given, subsequently the weight was increased until a failed attempt occurred. The 1RM was attained within five attempts.

#### $\dot {V}$O_2peak_

Following a 30 min rest, a continuously ramped $\dot {V}$O_2peak_ test was performed on an electrically braked cycle ergometer (Lode Excalibur, Groningen, The Netherlands). After a 5 min warm up at 30 W (females) or 50 W (males), workload progressively increased at 16 W min^−1^ until the participant reached volitional exhaustion. Oxygen consumption ($\dot {V}$O_2_) was obtained through breath-by-breath sampling (Cortex MetaLyzer 3B, Leipzig, Germany) that was calibrated prior to each test using gases of known concentrations (17.10% O_2_ and 5% CO_2_) and a 3 L Hans Rudolph syringe. $\dot {V}$O_2peak_ was determined as the highest value achieved over an 11 breath average. Heart rate was continuously recorded during the exercise (RS300, Polar, Finland) and participants were asked to maintain a cadence between 80 and 100 r·min^−1^.

### Diet and physical activity control

Participants were instructed to avoid alcohol and caffeine during the 48 h period prior to the two main experimental trials and the 96 h follow-up visit. A physical activity diary and weighed food diary was recorded 48 and 24 h before and throughout the first experimental trial, respectively. Participants were asked to replicate both physical activity levels and diet prior to each visit in the second experimental trial.

### Experimental trials

On the morning of each trial, participants arrived at laboratory at 0800 following an overnight fast (~ 10 h). A resting skeletal muscle biopsy sample was obtained from the middle portion of the vastus lateralis muscle of one leg. The participants then performed either the RE or RE + HIIT session. During both exercise sessions, participants received continuous verbal encouragement. For all trials, rating of perceived exertion (RPE, Category-Ratio 10 Scale) (Borg 1998) was recorded after each set of leg extensions and each 1 min bout of high-intensity cycling. Participants were allowed to consume water ad libitum throughout. Following the exercise session, the participant was free to leave and asked to return 4 days later after an overnight fast (~ 10 h) for a subsequent muscle biopsy taken 96 h after the RE component.

### Resistance exercise (RE) protocol

Participants completed a standardised warm up consisting of 2 sets of 8 repetitions of unilateral leg extensions at 30% 1RM, immediately followed by the contralateral leg. This was followed by 8 sets of 8 repetitions at 70% 1RM on each leg. Constant feedback and visual markers were provided in an attempt to match all repetitions for velocity (2-s concentric and eccentric phases) and range. Each set was separated by a 2 min recovery.

### Concurrent resistance exercise and high-intensity interval training (RE + HIIT) protocol

Immediately after an RE protocol identical to that described above, participants completed a 3 min warm up at 30–50 W on the cycle ergometer. This was followed by the completion of ten repetitions of 1 min cycling at an intensity designed to elicit 90% of their heart rate maximum (HR_max_), with each repetition separated by 1 min of cycling at 30 W (females) or 50 W (males). Participants were instructed to maintain a cadence between 80 and 100 r·min^−1^ during each interval.

### Muscle biopsies

Skeletal muscle samples were obtained from the middle portion of the vastus lateralis muscle using a 5-mm Bergström needle (Dixons Surgical Instruments, Essex, UK) modified with suction. Four millilitres of local anaesthesia (1% lidocaine) was administrated into the skin and subcutaneous tissue above the muscle belly of the vastus lateralis. Upon excision of a specimen, any visible fat and/or connective tissue was removed and excess blood was blotted on filter paper. Immediately after samples were dissected into pieces that were either snap frozen in liquid nitrogen, or embedded in Tissue-Tek optimum cutting temperature (OCT) compound (Agar Scientific, Essex, UK), immersed in liquid nitrogen-cooled isopentane, and stored at −80 °C until later analysis.

### Immunofluorescence microscopy

Muscle cross section (9 μm) from OCT-embedded tissue were cut at −20 °C using a cryostat microtome (Thermo Scientific, Runcorn, UK), and allowed to air dry for 30 min at room temperature, before being stored at −80 °C until subsequent analysis. Tissue sections were fixed in 4% paraformaldehyde fixing solution for 10 min at room temperature, washed with phosphate-buffered saline (PBS) containing 1% Tween-20 (PBST) for 3 × 5 min, and then blocked in PBS containing 2% bovine serum albumin, 2% goat serum, and 0.2% Triton X-100 for 60 min at room temperature. After blocking, sections were incubated with the primary antibodies diluted in PBS blocking solution overnight. Samples were then washed in PBST for 3 × 5 min before secondary antibodies diluted in PSB block were applied and incubated for 2 h. Subsequently, samples were washed in PBST for 4 × 5 min and then covered with a drop of Fluoromount™ aqueous mounting medium (Sigma–Aldrich, Dorset, UK) and a cover slip, and stored in the dark at 4 °C until viewing.

Two serial cross sections were stained; (1) for satellite cell content [paired box transcription factor 7 (Pax7), laminin and 4,6-diamidino-2-phenylindole dihydrochloride (DAPI)], and (2) for the number of active satellite cells [myogenic differentiation 1 (MyoD), laminin and DAPI]. Mouse anti-human antibodies directed against Pax7 [1:200; Developmental Studies Hybridoma Bank (DSHB), Iowa City, IA, USA] and MyoD (clone 5.8A; M351201-2; 1:50; Dako, Burlington, ON, Canada) with goat anti-mouse Alexa Fluor 488-conjugate IgG (A11029; 1:500; Invitrogen, Paisley, UK) secondary antibodies were used to detect quiescent and active satellite cells, respectively. All slides were counterstained with rabbit anti-human antibodies directed against laminin (AB11575; 1:500; Abcam, Cambridge, MA, USA) with goat anti-rabbit Alexa Fluor 647-conjugate IgG (A21245; 1:1000; Invitrogen) secondary antibodies to detect cell border, and DAPI (F4680; 1:20,000; Sigma–Aldrich) to reveal myonuclei.

Double staining of all samples was performed to determine muscle fiber type-specific localisation of satellite cells. Samples were washed in PBST for 4 × 5 min, re-fixed, and blocked. Mouse anti-human antibodies directed against myosin heavy chain I (MHC I; A4.951; 1:1000; DSHB) with goat anti-mouse Alexa Fluor 488-conjugate IgG secondary antibodies were used to detect type I muscle fiber isoforms. Rabbit anti-human antibodies directed against MHC II (AB91506; 1:2000; Abcam) with goat anti-mouse Alexa Fluor 647-conjugate IgG secondary antibodies were used to detect type II muscle fiber isoforms.

### Imaging and quantification

Images were viewed at 20× magnification (Leica DM2500, Leica Microsystems, Milton Keynes, UK) and captured with a digital camera (Leica DFC360 FX, Leica Microsystems). At least 50 type I and 75 type II muscle fibers were counted to ensure accurate assessment of the muscle fiber-specific satellite cell content (Mackey et al. 2009). In this study, 63 ± 4 type I and 92 ± 6 type II muscle fibers were evaluated for each muscle biopsy per participant. Image processing and quantitative analyses were completed using ImageJ version 1.49 software (Schneider et al. 2012). All quantitative analyses were conducted in a blinded fashion to the participant coding and experimental trial. The identification of Pax7^+^ and MyoD^+^ cells were determined by the co-localisation of either Pax7 or MyoD with DAPI, and located at the periphery of each muscle fiber. Double stained muscle cross sections (first stained for satellite cell and then fiber type) were superimposed to determine the satellite cell response in a muscle fiber-specific manner. This is important as previous studies have shown changes in RE-induced satellite cell response occur in a muscle fiber type-specific manner (Snijders et al. 2012; Cermak et al. 2013).

### RNA extraction and reverse transcription

Skeletal muscles samples were homogenised at 20 Hz for 2 × 3 min using a TissueLyser II (Qiagen, Limbury, Netherlands) in 1.0 mL of ice-cooled TRI Reagent (Sigma–Aldrich). Following centrifugation at 13,000×*g* for 15 min at 4 °C the supernatant was incubated for 5 min at room temperature. Next, 200 µL of chloroform was added and mixed for 20 s then allowed to stand for a further 10 min at room temperature before centrifugation. The upper, clear, aqueous phase containing total RNA was mixed with one volume of isopropanol and incubated for 30 min at room temperature before further centrifugation. The RNA pellet was washed in 1.0 mL of ice-cooled 70% ethanol, centrifuged at 7,500×*g* for 5 min and then repeated, before air-drying. Precipitated RNA was then re-suspended in nuclease-free water. One microliter of each RNA sample was analysed on a NanoDrop 2000 UV–Vis Spectrophotometer (Thermo Scientific, Rockford, IL, USA) to determine RNA concentration and purity. RNA concentration was 166.1 ± 18.3 ng·µL^−1^, and the *A*_260_/*A*_280_ ratio, as a measure of purity was 1.86 ± 0.06. An Agilent 210 Expert Bioanalyser with RNA 6000 Nano LabChip kits (Agilent Technologies, Palo Alto, CA, USA) was used to analyse the size and distribution of extracted RNA molecules. An RNA Integrity Number (RIN) was calculated for all samples based on the RIN algorithm of the Agilent 2100 Expert software (version B.02.08). The RIN was 6.6 ± 0.2. Reverse transcription of 20 µL of cDNA was performed using 1 µg of RNA with a high-capacity RNA-to-cDNA kit (Invitrogen). The cDNA samples were then stored at −20 °C until further analysis.

### Real-time quantitative polymerase chain reaction (PCR)

Real-time quantitative PCR was performed on a ViiA 7 real-time PCR system (Applied Biosystems, Forest City, CA, USA) under the following PCR cycle conditions; 50 °C for 2 min + 95 °C for 10 min + [(95 °C for 15 s + 60 °C for 1 min) × 40 cycles]. PCR reactions with 2 × TaqMan Universal Master Mix II with UNG (Invitrogen) and 20 × TaqMan Gene Expression assays (Invitrogen) according to the manufacturer’s instructions were used to determine mRNA expression levels for myogenic differentiation 1 (MyoD1, Hs00159528_m1), myogenic factor 5 (Myf5, hs00929416_g1), myogenin (MyoG, Hs00231167_m1), myogenic factor 6 (Myf6, Hs01547104_g1), myostatin (Hs00976237_m1) and β-2-microglobulin (β2M, Hs00984230_m1). In addition, PCR reactions with 2× SYBR Green JumpStart Taq Ready Mix (Sigma–Aldrich), forward and reverse primers (Sigma–Aldrich) at 500 nmol·L^−1^ were used to determine the mRNA expression levels for β-actin (Primer Design, Southampton, UK) and DNA topoisomerase 1 (TOP1, Primer Design). A melt curve was run on all SYBR Green PCR reactions to assess the amplification specificity. All samples were run in triplicate, and all samples from each participant were run together on the same plate to allow for relative comparison. Data were analysed by cycle threshold values, calculating relative expression using the 2^−∆∆CT^ method. Gene expression was normalised using the geometric mean of three reference genes (β2M, β-actin, TOP1).

### Statistical analysis

Data were analysed using IBM SPSS version 22 statistical software (IBM Corp., Armonk, NY, USA). All outcomes were examined using linear mixed models with repeated-measures and each participant as a random effect. This statistical model allows use of all available data, while avoiding imputation of missing data (RE at baseline in one male participant). A linear mixed model was used to examine differences in RPE responses with exercise trial included as a fixed factor. Changes in satellite cell content (Pax7^+^ cells), active satellite cell number (MyoD^+^ cells), mRNA expression and muscle fiber type distribution were analysed using a linear mixed model with time and exercise trial included as fixed effects. Muscle fiber types were analysed separately. When an interaction was identified a pairwise multiple comparisons with a Bonferroni correction was used to locate differences. Differences in all data sets were considered statistically significant at a two-tailed critical level of *P* < 0.05. Data are expressed as mean ± standard error of mean (SEM). A *priori* sample size calculation was performed using G*Power software (Version 3.1.7; Faul et al. 2007). Based on previously published data (Babcock et al. 2012), it was determined that a sample size of six participants would be necessary. This sample size would allow detection of a mean change of 0.024 in satellite cell content (Pax7^+^) per muscle fiber. Sample size calculation was performed with an alpha error of 0.05, and a power of 80%.

## Results

### Exercise trial responses

All participants completed the same number of sets and repetitions (8 sets × 8 repetitions at 70% 1RM). The RE workload was 38.8 ± 2.0 kg for males and 21.3 ± 2.5 kg for females. The HIIT workload during the 60 s effort was 245 ± 38 W for males and 120 ± 16 W for females. Heart rate during HIIT intervals corresponded to 90 ± 2% of HR_max_. No differences for RPE scores (all *P* ≥ 0.11) were observed between exercise components in both trials with RE and RE + HIIT being rated as equally strenuous [RE only, 6.2 ± 0.6; RE + HIIT, 6.2 ± 0.6; (RE component, 6.7 ± 0.6; HIIT component, 5.6 ± 0.5)].

### Muscle fiber characteristics

Muscle fiber composition was 40.4 ± 2.6% type I and 59.6 ± 2.5% type II muscle fibers. There were no statistical differences observed in fiber type distribution between trials or across time (all *P* ≥ 0.10).

### Satellite cell content (Pax7^+^ cells)

**Fig. 3:**
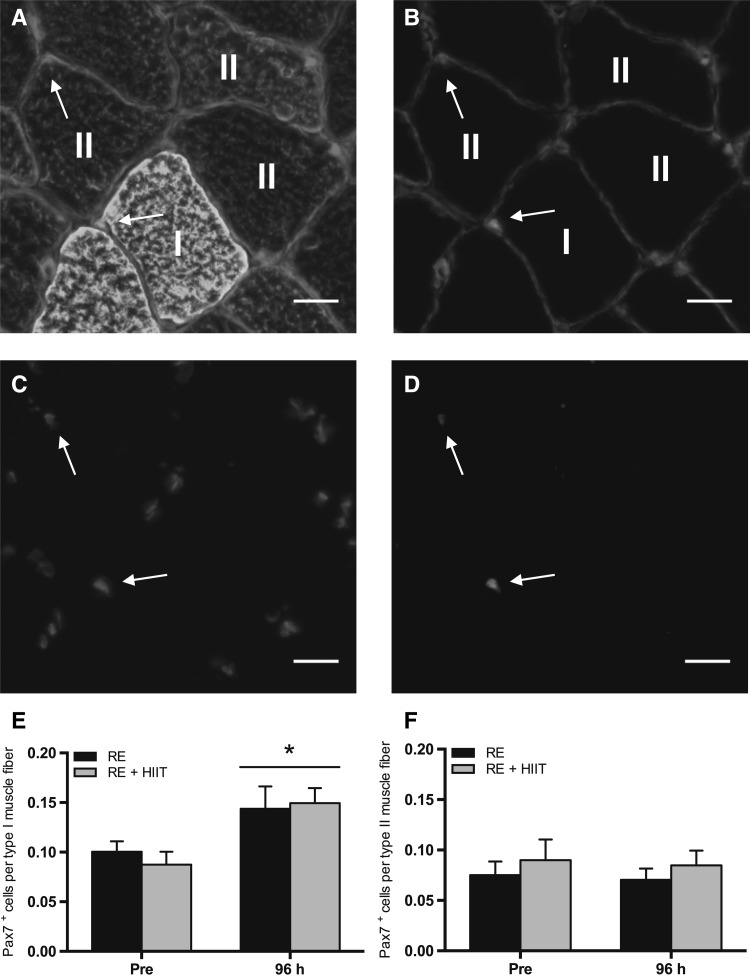
Satellite cell content (Pax7^+^) before and 96 h after a single bout of resistance exercise (RE) versus resistance exercise and high-intensity interval training (RE + HIIT). **a**–**d** Representative images of muscle fiber type-specific Pax7 immunofluorescent staining. Merged images of **a** Pax7/DAPI/laminin/MHC I (green)/MHC II (red), and **b** Pax7/DAPI/laminin (red) are provided, with single channel views of **c** DAPI (blue) and **d** Pax7 (green). Arrow denotes a Pax7^+^ cell. Scale bar 20 µm. Pax7^+^ cells per **e** type I and **f** type II muscle fiber before and 96 h after resistance exercise in both trials. Symbols above lines denote differences when a main effect was observed. *P < 0.05 vs. Pre. Data presented as mean ± SEM. (Color figure online)

Representative immunofluorescent images are shown in Fig. 3a–d. There was a main effect of time (*P* = 0.001), but no main effect of trial (*P* = 0.73), or an interaction effect (*P* = 0.45) for satellite cell content (Pax7^+^ cells) per type I muscle fiber (Fig. 3e). Muscle fiber type-I-specific satellite cell content increased (78 ± 24%) at 96 h compared to baseline following both exercise protocol. There were no main effects of time (*P* = 0.71), trial (*P* = 0.36), or an interaction effect (*P* = 0.98) in satellite cell content per type II muscle fiber (Fig. 3f).

### Number of active satellite cells (MyoD^+^ cells)

**Fig. 4:**
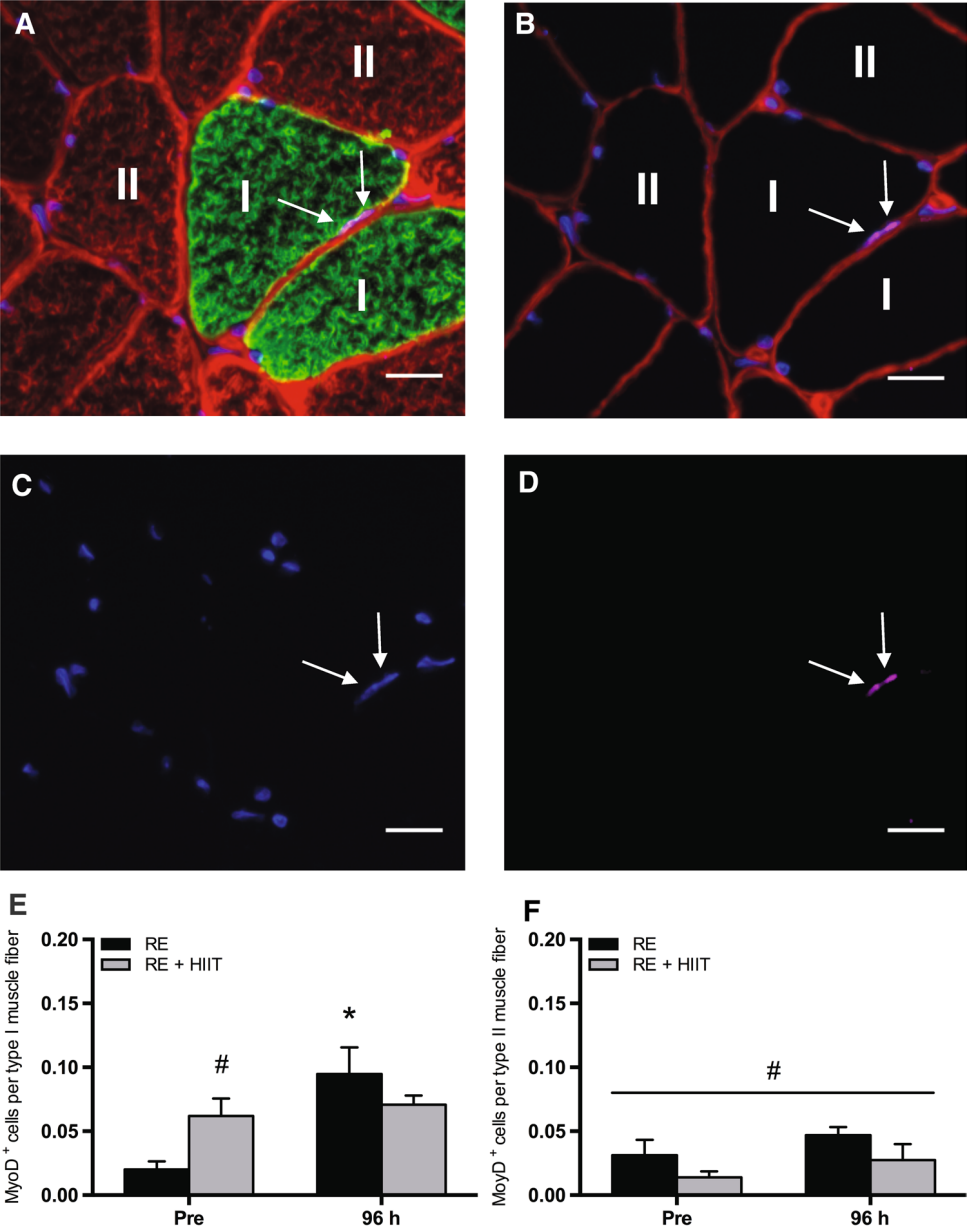
Number of active satellite cells (MyoD^+^ cells) before and 96 h after a single bout of resistance exercise (RE) versus resistance exercise and high-intensity interval training (RE + HIIT). **a**–**d** Representative images of muscle fiber type-specific MyoD immunofluorescent staining. Merged images of **a** MyoD/DAPI/laminin/MHC I (green)/MHC II (red), and **b** MyoD/DAPI/laminin (red) are provided, with single channel views of **c** DAPI (blue) and **d** MyoD (purple). Arrow denotes a MyoD^+^ cell. Scale bar 20 µm. MyoD^+^ cells per **e** type I and **f** type II muscle fiber before and 96 h after resistance exercise in both trials. Symbols above lines denote differences when a main effect was observed. Symbols without lines denote differences revealed by a post-hoc test when an interaction effect was observed. *P < 0.05 vs. Pre; ^#^ *P* < 0.05 vs. RE. Data presented as mean ± SEM. (Color figure online)

Representative immunofluorescent images are shown in Fig. 4a–d. There was a main effect of time (*P* = 0.006) and an interaction effect (*P* = 0.025), but no main effect of trial (*P* = 0.53), in the number of active satellite cells (MyoD^+^ cell) per type I muscle fiber (Fig. 4e). Post hoc analysis revealed an increase (*P* = 0.001) in muscle fiber type-I-specific MyoD^+^ cells at 96 h compared to baseline following RE. Conversely, muscle fiber type-I-specific MyoD^+^ cells remained unchanged over time for RE + HIIT (*P* = 0.64). There was no difference (*P* = 0.21) between exercise trials at 96 h. However, RE + HIIT demonstrated an elevated (*P* = 0.046) baseline value compared to RE, which may have impacted the RE + HIIT exercise response. There was a main effect of trial (*P* = 0.049), but no main effect of time (*P* = 0.13), or an interaction effect (*P* = 0.96) in MyoD^+^ cell per type II muscle fiber (Fig. 4f). There was an overall higher number of MyoD^+^ cell per type II muscle fiber in RE as compared to RE + HIIT.

### Intramuscular mRNA expression

**Fig. 5:**
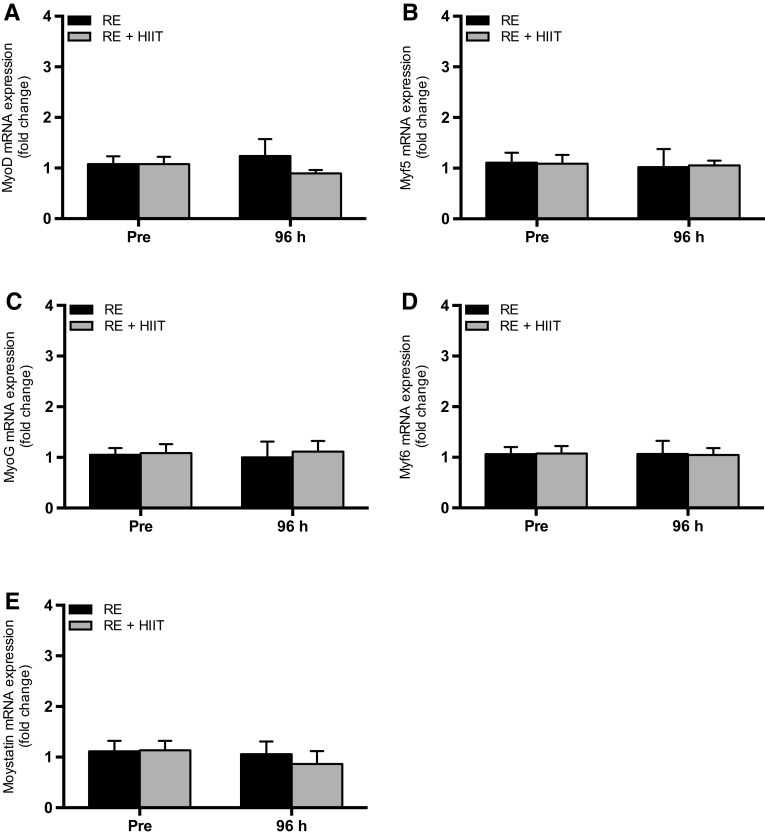
mRNA expression of **a** MyoD, **b** Myf5, **c** MyoG, **d** Myf6 and **e** myostatin before and 96 h after a single bout of resistance exercise (RE) versus resistance exercise and high-intensity interval training (RE + HIIT). Data presented as mean ± SEM

There were no main effects of time (all *P* ≥ 0.49), trial (all *P* ≥ 0.38) or an interaction effect (all *P* ≥ 0.39) for the expression of MyoD, Myf5, MyoG, Myf6 and myostatin mRNA (Fig. 5).

## Discussion

The aim of this study was to establish the effect of a single bout of concurrent RE + HIIT compared to an isolated RE session on the total and active number of satellite cells at rest and 4 days (96 h) after exercise in sedentary, overweight/obese, middle-aged individuals. For the first time, it is shown that both a single bout of RE and concurrent RE + HIIT results in an increase in satellite cell content in type-I-specific muscle fibers, with no difference between exercise regimens. In addition, there was no difference in the number of active (MyoD^+^ cells) type-I-specific satellite cells at 96 h after both exercise trials. The current findings imply that concurrent RE + HIIT does not compromise the transient RE-induced increase in total satellite cell content, and possibly the number of active satellite cells, after 96 h in sedentary, overweight/obese, middle-aged individuals. Therefore, concurrent RE + HIIT exercise programmes may offer a potent, time-efficient exercise strategy, which could help those that may not be “natural exercisers” meet the current exercise guidelines.

Expansion in satellite cell content peaks between 72 and 96 h following exercise, and declines thereafter (Martin and Lewis 2012; Snijders et al. 2015). The present study demonstrated a 78 ± 24% increase in the satellite cell content associated with type I muscle fibers 96 h after both exercise protocols. While previous exercise protocols using higher workloads have elicited greater increases (> 95%) in satellite cell content (Crameri et al. 2004; O’Reilly et al. 2008; McKay et al. 2009; Mikkelsen et al. 2009), the 78 ± 24% increase in the present study is comparable with data where similar workloads were used (Babcock et al. 2012; Snijders et al. 2014). The RE used in the current study was designed to represent a realistic, exercise program for untrained and overweight/obese individuals, and therefore, characterises a real-life exercise-induced stimulus.

Consistent with the present study, an expansion in satellite cell content following a single bout of RE has been reported in both young and older adults (Martin and Lewis 2012; Snijders et al. 2015), and at least in the young, has been shown to be important in determining changes in muscle mass to chronic exercise training (Bellamy et al. 2014). While it remains to be determined if this is true in middle-aged and older adults, an exercise program resulting in a potent expansion of satellite cell content is likely to help reduce muscle mass loss with ageing. The satellite cell content associated with type I fibers has been shown to acutely increase following RE in older adults. However, in type II fibers this response is blunted (Snijders et al. 2014), or fails to respond entirely (McKay et al. 2012; Nederveen et al. 2015). An attenuated decline in myostatin colocalisation with satellite cells has been proposed as one mechanism for this blunted response in older adults (McKay et al. 2012; Snijders et al. 2014). Similarly, the current study demonstrates that both exercise programs resulted in an increase in type I associated satellite cell content, but no expansion in the satellite cell content was noted with type II fibers in sedentary, overweight/obese, middle-aged individuals. It should be noted that the lack of satellite cell response in type II muscle fibers could merely be reflective of the timing of the final biopsy.

The data from the current study suggest that concurrent RE + HIIT does not impair the elevation in satellite cell content, particularly in type I fibers, 96 h after a single bout of RE, which is in contrast to a similar study that employed moderate-intensity continuous endurance exercise instead of HIIT (Babcock et al. 2012). However, rodent studies have suggested that the increase in satellite cell content is related to exercise intensity rather than duration (Kurosaka et al. 2012). In humans, two recent studies implementing similar HIIT protocols to the current study have demonstrated that HIIT could offer a greater hypertrophic stimulus than moderate-intensity endurance exercise in sedentary older men (Bell et al. 2015; Nederveen et al. 2015). Similarly, an increase in lean mass in leg and groin regions has been observed following 6 weeks of HIIT in overweight women (Gillen et al. 2013). The anabolic potential of HIIT, therefore, raises the hypothesis that incorporating HIIT, rather than moderate-intensity continuous endurance exercise, concurrently with RE, may act to abolish the interference effect between the different adaptive responses.

The satellite cell data is supported by that of the myogenic regulatory factor (MRF) MyoD, which represents an important marker of satellite cell activity. MyoD is expressed during activation, proliferation and during the early stages of differentiation, but not in quiescent satellite cells (Zammit et al. 2004). In the present study, the increase in the number of MyoD^+^ cells is similar to that previously reported after a single bout of RE or HIIT (Nederveen et al. 2015). Specifically, the type I associated MyoD^+^ cell number showed no difference between exercise trials at 96 h, reflecting the increase in satellite cell content at 96 h in both exercise trials. However, no change in type I associated MyoD^+^ cell number was reported from baseline to 96 h in RE + HIIT, whereas RE demonstrated an increase. This is likely due to the reported high baseline value in RE + HIIT compared to RE, which may have masked any change from baseline. The reason for this difference at baseline is unknown. Furthermore, the reason for the overall difference in muscle fiber type-II-specific MyoD^+^ cell number between exercise trials is unknown. Irrespective of this, muscle fiber type-II-specific MyoD^+^ cell number remained unchanged across time in both exercise trials with no evidence of an interference of concurrent RE + HIIT. It is worth noting that in the current study there were no significant differences in satellite cell content following RE or RE + HIIT despite a greater workload completed in the concurrent exercise trial. While this study clearly demonstrates that RE + HIIT does not affect the RE-induced satellite cell response, it does raise the question as to whether the magnitude of the satellite cell response could be further increased if the concurrent exercise strategy is optimised.

Myostatin is known to be a negative regulator of muscle growth (McPherron and Lee 1997; Reisz-Porszasz et al. 2003) through the suppression of muscle protein synthesis and satellite cell activity (Langley et al. 2002; McCroskery et al. 2003; Welle et al. 2009). A single bout of RE or endurance exercise, either in isolation or in combination, has been shown to decrease myostatin mRNA expression (Louis et al. 2007; Lundberg et al. 2012). While the present study showed no statistical change in myostatin mRNA expression after either exercise trial, we have previously demonstrated a downregulation in myostatin mRNA after exercise (< 6 h) with no differences between RE and RE + HIIT (Pugh et al. 2015). The lack of consistency could be explained by timing of the muscle biopsy. No changes in mRNA expression of the MRFs (MyoD, Myf5, MyoG and Myf6) were found at 96 h compared to baseline after either exercise trial. Others have reported increases in MyoG and Myf6 mRNA expression up to 120 h after high-volume, muscle damaging RE (McKay et al. 2008). However, it is likely that less damaging exercise results in earlier (< 96 h) peaks in mRNA expression of MRFs (Psilander et al. 2003; Yang et al. 2005). Whilst the present study shows no change in MyoD mRNA expression, the increase in MyoD^+^ cells may suggest that the increases in transcription have already occurred within the 96 h timeframe. Future studies are warranted to investigate the temporal response of myostatin and MRFs expression, both at the gene transcription and protein level, on the effect of satellite cell regulation in response to concurrent exercise.

Given that HIIT improves the satellite cell response, but also has profound health benefits across both healthy (Trapp et al. 2008; Gillen et al. 2013; Faulkner et al. 2015) and clinical populations (Currie et al. 2013; Madsen et al. 2015; Cassidy et al. 2016) matching or exceeding that of traditional endurance exercise (Tjønna et al. 2008; Kemmler et al. 2015), it would suggest that this form of higher intensity activity should be the preferred option for concurrent exercise regimens. In addition, HIIT has also been described as a time-efficient alternative (< 25 min) to more traditional moderate-intensity exercise (Gibala et al. 2012), with reports of high exercise adherence (Terada et al. 2013; Currie et al. 2013; Faulkner et al. 2015) and enjoyment (Bartlett et al. 2011). The data from the present study indicates that incorporating HIIT after RE does not dampen the increase in satellite cell content following a single bout of RE, while potentially providing the stimulus for important cardiovascular and metabolic adaptations previously attributed to HIIT (Gibala et al. 2012). However, acute exercise studies only provide a framework, and therefore, further work into the long-term consequences of chronic concurrent RE and HIIT on the satellite cell response compared to RE training only is warranted.

### Practical implications of concurrent training

Often a lack of time is cited as the main barrier to an individual participating in regular physical activity (Stutts 2002; Trost et al. 2002). This is particularly true when both RE and endurance exercise components are required. Scheduling each exercise component to occur within a single session, such as with concurrent training, has been shown to be the preferred option for individuals with type 2 diabetes (Larose et al. 2012). This illustrates the importance of minimising the number of training visits, together with overall time commitment. Specifically, the concurrent RE + HIIT program used here was completed within a single exposure minimising both the exercise time commitment (HIIT vs. moderate-intensity continuous endurance exercise: 75 vs. 150 min), and the number of training sessions per week (concurrent RE + HIIT vs. individual exercise sessions: three vs. six sessions). This study has demonstrated the feasibility of this concurrent RE + HIIT model in sedentary, overweight/obese, middle-aged individuals, which may provide these individuals with an alternative strategy to increase their regular physical activity levels. However, future studies are warranted to determine the chronic effectiveness of this concurrent RE + HIIT program.

### Limitations of the study

A potential limitation of the study is that the total work executed was higher in RE + HIIT compared to RE. It is plausible that any interference effect could have been masked with the results reflecting differences in contractile activity, rather than the exercise-mode (i.e., HIIT). However, this study set out to examine the interference effect to a practical/realistic exercise model that could be applied to the general population, and therefore, a prolonged RE-only session would not have been appropriate. Additionally, the sample size in the current study is limited. However, the data indicate clear beneficial effects of concurrent training on acute satellite cell function despite such a small sample size. Finally, in the current study there were different proportions of men and women, which may have led to gender bias. No statistical analysis was performed for sex differences in the present study due to a limited sample size. However, descriptively there were no gender effects. Similarly, others have shown no gender effects when using a mixed gender model (Fry et al. 2014).

## Conclusion

For the first time, we have shown that concurrent RE + HIIT does not inhibit the increase in the satellite cell content, and possibly active satellite cell number, arising from a single bout of RE. Concurrent RE + HIIT may offer a time-efficient way to maximise the physiological benefits from a single bout of exercise in sedentary, overweight/obese, middle-aged individuals. Future studies are warranted to determine if long-term concurrent RE + HIIT affect muscle strength and growth adaptations compared to RE training in isolation.

## Abbreviations

1RM: One-repetition maximum
BMI: Body mass index (kg·m^2^)
HIIT: High-intensity interval training
HR_max_: Heart rate maximum
Myf5: Myogenic factor 5
Myf6: Myogenic factor 6
MyoD: Myogenic differentiation 1
MyoG: Myogenin
Pax7: Paired box transcription factor 7
RE: Resistance exercise
RE + HIIT: Concurrent resistance exercise and high-intensity interval training
RPE: Rate of perceived exertion
SEM: Standard error of mean
$\dot {V}$O_2 peak_: Peak oxygen uptake (mL·kg^−1^·min^−1^)
W: Watts (W)

## References

[CR1] Alberti KGMM, Zimmet P, Shaw J (2005). The metabolic syndrome—a new worldwide definition. Lancet.

[CR2] Apró W, Wang L, Pontén M, Blomstrand E, Sahlin K (2013). Resistance exercise induced mTORC1 signaling is not impaired by subsequent endurance exercise in human skeletal muscle. Am J Physiol Endocrinol Metab.

[CR3] Babcock L, Escano M, D’Lugos A, Todd K, Murach K, Luden N (2012). Concurrent aerobic exercise interferes with the satellite cell response to acute resistance exercise. Am J Physiol Regul Integr Comp Physiol.

[CR4] Bartlett JD, Close GL, MacLaren DPM, Gregson W, Drust B, Morton JP (2011). High-intensity interval running is perceived to be more enjoyable than moderate-intensity continuous exercise: implications for exercise adherence. J Sports Sci.

[CR5] Bell GJ, Petersen SR, Wessel J, Bagnall K, Quinney HA (1991). Physiological adaptations to concurrent endurance training and low velocity resistance training. Int J Sports Med.

[CR6] Bell KE, Séguin C, Parise G, Baker SK, Phillips SM (2015). Day-to-day changes in muscle protein synthesis in recovery from resistance, aerobic, and high-intensity interval exercise in older men. J Gerontol Ser A Biol Sci Med Sci.

[CR7] Bellamy LM, Joanisse S, Grubb A, Mitchell CJ, McKay BR, Phillips SM, Baker S, Parise G (2014). The acute satellite cell response and skeletal muscle hypertrophy following resistance training. PLoS One.

[CR8] Bijlsma AY, Meskers CGM, Ling CHY, Narici M, Kurrle SE, Cameron ID, Westendorp RGJ, Maier AB (2013). Defining sarcopenia: the impact of different diagnostic criteria on the prevalence of sarcopenia in a large middle aged cohort. Age (Dordr).

[CR9] Borg G (1998). Borg’s perceived exertion and pain scales.

[CR10] Cassidy S, Thoma C, Hallsworth K, Parikh J, Hollingsworth KG, Taylor R, Jakovljevic DG, Trenell MI (2016). High intensity intermittent exercise improves cardiac structure and function and reduces liver fat in patients with type 2 diabetes: a randomised controlled trial. Diabetologia.

[CR11] Cermak NM, Snijders T, McKay BR, Parise G, Verdijk LB, Tarnopolsky MA, Gibala MJ, van Loon LJC (2013). Eccentric exercise increases satellite cell content in type II muscle fibers. Med Sci Sport Exerc.

[CR12] Charifi N, Kadi F, Féasson L, Denis C (2003). Effects of endurance training on satellite cell frequency in skeletal muscle of old men. Muscle Nerve.

[CR13] Chief Medical Office (2011) Physical activity guidelines in the UK: review and recommendations

[CR14] Coffey VG, Jemiolo B, Edge J, Garnham AP, Trappe SW, Hawley JA (2009). Effect of consecutive repeated sprint and resistance exercise bouts on acute adaptive responses in human skeletal muscle. Am J Physiol Regul Integr Comp Physiol.

[CR15] Coffey VG, Pilegaard H, Garnham AP, O’Brien BJ, Hawley JA (2009). Consecutive bouts of diverse contractile activity alter acute responses in human skeletal muscle. J Appl Physiol.

[CR16] Craig B, Lucas J, Pohlman R, Stelling H (1991). The effects of running, weightlifting and a combination of both on growth hormone release. J Appl Sport Sci Res.

[CR17] Crameri RM, Langberg H, Magnusson P, Jensen CH, Schrøder HD, Olesen JL, Suetta C, Teisner B, Kjaer M (2004). Changes in satellite cells in human skeletal muscle after a single bout of high intensity exercise. J Physiol.

[CR18] Crameri RM, Aagaard P, Qvortrup K, Langberg H, Olesen J, Kjaer M (2007). Myofibre damage in human skeletal muscle: effects of electrical stimulation versus voluntary contraction. J Physiol.

[CR19] Currie KD, Dubberley JB, McKelvie RS, Macdonald MJ (2013). Low-volume, high-intensity interval training in patients with CAD. Med Sci Sports Exerc.

[CR20] Dominguez LJ, Barbagallo M (2007). The cardiometabolic syndrome and sarcopenic obesity in older persons. J Cardiometab Syndr.

[CR21] Donges CE, Burd NA, Duffield R, Smith GC, West DWD, Short MJ, Mackenzie R, Plank LD, Shepherd PR, Phillips SM, Edge JA (2012). Concurrent resistance and aerobic exercise stimulates both myofibrillar and mitochondrial protein synthesis in sedentary middle-aged men. J Appl Physiol.

[CR22] Dreyer HC, Blanco CE, Sattler FR, Schroeder ET, Wiswell RA (2006). Satellite cell numbers in young and older men 24 h after eccentric exercise. Muscle Nerve.

[CR23] Faul F, Erdfelder E, Lang A-G, Buchner A (2007). G*Power 3: a flexible statistical power analysis program for the social, behavioral, and biomedical sciences. Behav Res Methods.

[CR24] Faulkner S, Pugh J, Hood T, Menon K, King J, Nimmo M (2015). Group studio cycling; an effective intervention to improve cardio-metabolic health in overweight physically inactive individuals. J Fit Res.

[CR80] Fry CS, Noehren B, Mula ZJ, Ubele MF, Westgate PM, Kern PA, Peterson CA (2014). Fibre type-specific satellite cell response to aerobic training in sedentary adults. J Physiol.

[CR25] Fyfe JJ, Bartlett JD, Hanson ED, Stepto NK, Bishop DJ (2016). Endurance training intensity does not mediate interference to maximal lower-body strength gain during short-term concurrent training. Front Physiol.

[CR26] Garber CE, Blissmer B, Deschenes MR, Franklin BA, Lamonte MJ, Lee I-M, Nieman DC, Swain DP (2011). Quantity and quality of exercise for developing and maintaining cardiorespiratory, musculoskeletal, and neuromotor fitness in apparently healthy adults: guidance for prescribing exercise. Med Sci Sports Exerc.

[CR27] Gibala MJ, Little JP, Macdonald MJ, Hawley JA (2012). Physiological adaptations to low-volume, high-intensity interval training in health and disease. J Physiol.

[CR28] Gillen JB, Percival ME, Ludzki A, Tarnopolsky MA, Gibala MJ (2013). Interval training in the fed or fasted state improves body composition and muscle oxidative capacity in overweight women. Obesity.

[CR29] Hamilton DL, Philp A (2013). Can AMPK mediated suppression of mTORC1 explain the concurrent training effect?. Cell Mol Exerc Physiol.

[CR30] Health and Social Care Information Centre (2013) Health survey for England, 2012—health, social care and lifestyles: summary of key findings. https://digital.nhs.uk. Accessed 8 Sep 2016

[CR31] Health and Social Care Information Centre (2016) Statistics on obesity, physical activity and diet. https://digital.nhs.uk. Accessed 8 Sep 2016

[CR32] Hennessy L, Watson A (1994). The interference effects of training for strength and endurance simultaneously. J Strength Cond Res.

[CR33] Hickson RC (1980). Interference of strength development by simultaneously training for strength and endurance. Eur J Appl Physiol Occup Physiol.

[CR34] Jackson AS, Janssen I, Sui X, Church TS, Blair SN (2012). Longitudinal changes in body composition associated with healthy ageing: men, aged 20–96 years. Br J Nutr.

[CR35] Kazior Z, Willis SJ, Moberg M, Apró W, Calbet JAL, Holmberg HC, Blomstrand E (2016). Endurance exercise enhances the effect of strength training on muscle fiber size and protein expression of akt and mTOR. PLoS One.

[CR36] Kemmler W, Lell M, Scharf M, Fraunberger L, von Stengel S (2015). High versus moderate intense running exercise—effects on cardiometabolic risk-factors in untrained males. Dtsch Med Wochenschr.

[CR37] Kikuchi N, Yoshida S, Okuyama M, Nakazato K (2016). The effect of high-intensity interval cycling sprints subsequent to arm-curl exercise on upper-body muscle strength and hypertrophy. J Strength Cond Res.

[CR38] Kurosaka M, Naito H, Ogura Y, Machida S, Katamoto S (2012). Satellite cell pool enhancement in rat plantaris muscle by endurance training depends on intensity rather than duration. Acta Physiol (Oxf).

[CR39] Langley B, Thomas M, Bishop A, Sharma M, Gilmour S, Kambadur R (2002). Myostatin inhibits myoblast differentiation by down-regulating MyoD expression. J Biol Chem.

[CR40] Larose J, Sigal RJ, Khandwala F, Kenny GP (2012). Comparison of strength development with resistance training and combined exercise training in type 2 diabetes. Scand J Med Sci Sports.

[CR42] Louis E, Raue U, Yang Y, Jemiolo B, Trappe S (2007). Time course of proteolytic, cytokine, and myostatin gene expression after acute exercise in human skeletal muscle. J Appl Physiol.

[CR43] Lundberg TR, Fernandez-Gonzalo R, Gustafsson T, Tesch PA (2012). Aerobic exercise alters skeletal muscle molecular responses to resistance exercise. Med Sci Sports Exerc.

[CR44] Lundberg TR, Fernandez-Gonzalo R, Gustafsson T, Tesch PA (2013). Aerobic exercise does not compromise muscle hypertrophy response to short-term resistance training. J Appl Physiol.

[CR45] Mackey AL, Kjaer M, Charifi N, Henriksson J, Bojsen-Moller J, Holm L, Kadi F (2009). Assessment of satellite cell number and activity status in human skeletal muscle biopsies. Muscle Nerve.

[CR46] Madsen SM, Thorup AC, Overgaard K, Jeppesen PB (2015). High intensity interval training improves glycaemic control and pancreatic β cell function of type 2 diabetes patients. PLoS One.

[CR47] Marcell TJ (2003). Sarcopenia: causes, consequences, and preventions. J Gerontol Med Sci.

[CR48] Martin NR, Lewis MP (2012). Satellite cell activation and number following acute and chronic exercise: a mini review. Cell Mol Exerc Physiol.

[CR49] McCroskery S, Thomas M, Maxwell L, Sharma M, Kambadur R (2003). Myostatin negatively regulates satellite cell activation and self-renewal. J Cell Biol.

[CR50] McKay BR, O’Reilly CE, Phillips SM, Tarnopolsky MA, Parise G (2008). Co-expression of IGF-1 family members with myogenic regulatory factors following acute damaging muscle-lengthening contractions in humans. J Physiol.

[CR51] McKay BR, De Lisio M, Johnston APW, O’Reilly CE, Phillips SM, Tarnopolsky MA, Parise G (2009). Association of interleukin-6 signalling with the muscle stem cell response following muscle-lengthening contractions in humans. PLoS One.

[CR52] McKay BR, Ogborn DI, Bellamy LM, Tarnopolsky MA, Parise G (2012). Myostatin is associated with age-related human muscle stem cell dysfunction. FASEB J.

[CR53] McPherron AC, Lee SJ (1997). Double muscling in cattle due to mutations in the myostatin gene. Proc Natl Acad Sci USA.

[CR54] Mikkelsen UR, Langberg H, Helmark IC, Skovgaard D, Andersen LL, Kjaer M, Mackey AL (2009). Local NSAID infusion inhibits satellite cell proliferation in human skeletal muscle after eccentric exercise. J Appl Physiol.

[CR55] Nederveen JP, Joanisse S, Séguin CML, Bell KE, Baker SK, Phillips SM, Parise G (2015). The effect of exercise mode on the acute response of satellite cells in old men. Acta Physiol (Oxf).

[CR56] O’Reilly C, McKay B, Phillips S, Tarnopolsky M, Parise G (2008). Hepatocyte growth factor (HGF) and the satellite cell response following muscle lengthening contractions in humans. Muscle Nerve.

[CR57] Olesen J, Kiilerich K, Pilegaard H (2010). PGC-1alpha-mediated adaptations in skeletal muscle. Eur J Physiol.

[CR58] Psilander N, Damsgaard R, Pilegaard H (2003). Resistance exercise alters MRF and IGF-I mRNA content in human skeletal muscle. J Appl Physiol.

[CR59] Pugh JK, Faulkner SH, Jackson AP, King JA, Nimmo MA (2015). Acute molecular responses to concurrent resistance and high-intensity interval exercise in untrained skeletal muscle. Physiol Rep.

[CR60] Rana JS, Li TY, Manson JE, Hu FB (2007). Adiposity compared with physical inactivity and risk of type 2 diabetes in women. Diabetes Care.

[CR61] Reddigan JI, Ardern CI, Riddell MC, Kuk JL (2011). Relation of physical activity to cardiovascular disease mortality and the influence of cardiometabolic risk factors. Am J Cardiol.

[CR62] Reisz-Porszasz S, Bhasin S, Artaza JN, Shen R, Sinha-Hikim I, Hogue A, Fielder TJ, Gonzalez-Cadavid NF (2003). Lower skeletal muscle mass in male transgenic mice with muscle-specific overexpression of myostatin. Am J Physiol Endocrinol Metab.

[CR63] Schneider CA, Rasband WS, Eliceiri KW (2012). NIH Image to ImageJ: 25 years of image analysis. Nat Methods.

[CR64] Shaw BS, Shaw I, Brown GA (2009). Comparison of resistance and concurrent resistance and endurance training regimes in the development of strength. J Strength Cond Res.

[CR66] Snijders T, Verdijk LB, Hansen D, Dendale P, van Loon LJC (2011). Continuous endurance-type exercise training does not modulate satellite cell content in obese type 2 diabetes patients. Muscle Nerve.

[CR67] Snijders T, Verdijk LB, Beelen M, McKay BR, Parise G, Kadi F, van Loon LJC (2012). A single bout of exercise activates skeletal muscle satellite cells during subsequent overnight recovery. Exp Physiol.

[CR68] Snijders T, Verdijk LB, Smeets JSJ, McKay BR, Senden JMG, Hartgens F, Parise G, Greenhaff P, van Loon LJC (2014). The skeletal muscle satellite cell response to a single bout of resistance-type exercise is delayed with aging in men. Age (Dordr).

[CR69] Snijders T, Nederveen JP, McKay BR, Joanisse S, Verdijk LB, van Loon LJC, Parise G (2015). Satellite cells in human skeletal muscle plasticity. Front Physiol.

[CR70] Stenholm S, Harris T, Rantenen T, Visser M, Kritchevsky SB, Ferrucci L (2008). Sarcopenic obesity-definition,etiology and consequences. Curr Opin Clin Nutr Metab Care.

[CR71] Stutts WC (2002). Physical activity determinants in adults. Perceived benefits, barriers, and self efficacy. AAOHN J.

[CR72] Terada T, Friesen A, Chahal BS, Bell GJ, McCargar LJ, Boulé NG (2013). Feasibility and preliminary efficacy of high intensity interval training in type 2 diabetes. Diabetes Res Clin Pract.

[CR73] Tjønna AE, Lee SJ, Rognmo Ø, Stølen TO, Bye A, Haram PM, Loennechen JP, Al-Share QY, Skogvoll E, Slørdahl SA, Kemi OJ, Najjar SM, Wisløff U (2008). Aerobic interval training versus continuous moderate exercise as a treatment for the metabolic syndrome: a pilot study. Circulation.

[CR74] Trapp EG, Chisholm DJ, Freund J, Boutcher SH (2008). The effects of high-intensity intermittent exercise training on fat loss and fasting insulin levels of young women. Int J Obes (Lond).

[CR75] Trost SG, Owen N, Bauman AE, Sallis JF, Brown W (2002). Correlates of adults’ participation in physical activity: review and update. Med Sci Sports Exerc.

[CR76] Verney J, Kadi F, Charifi N, Féasson L, Saafi MA, Castells J, Piehl-Aulin K, Denis C (2008). Effects of combined lower body endurance and upper body resistance training on the satellite cell pool in elderly subjects. Muscle Nerve.

[CR77] Welle S, Burgess K, Mehta S (2009). Stimulation of skeletal muscle myofibrillar protein synthesis, p70 S6 kinase phosphorylation, and ribosomal protein S6 phosphorylation by inhibition of myostatin in mature mice. Am J Physiol Endocrinol Metab.

[CR78] Yang Y, Creer A, Jemiolo B, Trappe S (2005). Time course of myogenic and metabolic gene expression in response to acute exercise in human skeletal muscle. J Appl Physiol.

[CR79] Zammit PS, Golding JP, Nagata Y, Hudon V, Partridge TA, Beauchamp JR (2004). Muscle satellite cells adopt divergent fates: a mechanism for self-renewal?. J Cell Biol.

